# Diversity of symbioses between chemosynthetic bacteria and metazoans at the Guiness cold seep site (Gulf of Guinea, West Africa)

**DOI:** 10.1002/mbo3.47

**Published:** 2012-11-21

**Authors:** Sébastien Duperron, Clara F Rodrigues, Nelly Léger, Kamil Szafranski, Carole Decker, Karine Olu, Sylvie M Gaudron

**Affiliations:** 1 UMR 7138 (UPMC CNRS IRD MNHN), Systématique, Adaptation, Evolution, Université Pierre et Marie Curie7, quai St. Bernard, bâtiment A, 75005, Paris, France; 2 Departamento de Biologia and CESAM, Universidade de Aveiro, Campus Universitário de Santiago3810-193, Aveiro, Portugal; 3 Laboratoire Environnement Profond, Département Etudes des Ecosystèmes Profonds, Centre Ifremer de BrestBP 71, 29280, Plouzané, France

**Keywords:** *Acharax*, *Calyptogena*, chemosynthesis, cold seeps, *Elenaconcha*, Gulf of Guinea, *Lamellibrachia*, symbiosis, *Thyasira*

## Abstract

Fauna from deep-sea cold seeps worldwide is dominated by chemosymbiotic metazoans. Recently, investigation of new sites in the Gulf of Guinea yielded numerous new species for which symbiosis was strongly suspected. In this study, symbioses are characterized in five seep-specialist metazoans recently collected from the Guiness site located at ∼600 m depth. Four bivalve and one annelid species belonging to families previously documented to harbor chemosynthetic bacteria were investigated using bacterial marker gene sequencing, fluorescence in situ hybridization, and stable isotope analyses. Results support that all five species display chemosynthetic, sulfur-oxidizing γ-proteobacteria. Bacteria are abundant in the gills of bivalves, and in the trophosome of the siboglinid annelid. As observed for their relatives occurring at deeper sites, chemoautotrophy is a major source of carbon for animal nutrition. Although symbionts found in each host species are related to symbionts found in other metazoans from the same families, several incongruencies are observed among phylogenetic trees obtained from the different bacterial genes, suggesting a certain level of heterogeneity in symbiont strains present. Results provide new insights into the diversity, biogeography, and role of symbiotic bacteria in metazoans from the Gulf of Guinea, at a site located at an intermediate depth between the continental shelf and the deep sea.

## Introduction

Symbioses between chemosynthetic bacteria and metazoans are responsible for the high animal biomasses encountered at hydrothermal vents and cold seeps in the deep sea, because bacteria convert monocarbon compounds such as methane or dissolved inorganic carbon into complex organic molecules available to their hosts (Stewart et al. 2005; Dubilier et al. 2008). The importance of symbiosis is particularly evident when looking at huge biomasses reached by symbionts-associated metazoans flourishing around vents in the east Pacific Rise or at certain seep sites in the Gulf of Mexico (Van Dover 2000). Many other vent and seep sites around the world are less spectacular, and display much lower biomasses, but the fauna at these sites is still dominated by symbiont-associated animals, as seen at seeps in the eastern Mediterranean, Gulf of Cadiz, Norwegian, or Marmara Sea (Olu-LeRoy et al. 2004). At very shallow sites, however, this particular fauna is absent and replaced by animals deriving their carbon from photosynthetic primary production, with a limit approximately situated at 200 m depth for vents (Tarasov et al. 2005; Dando 2010). Shelf seeps are inhabited by a subset of typical shelf fauna, which includes the sulfide-tolerant species, and typical seep groups are often absent or rare, both in terms of biomass and species number (Levin et al. 2000). Chemosymbiotic species present at shallow sites are not the typical vent or seep-specialists, apart from a few exceptions such as the vent obligate *Siboglinum* (Bay of Plenty, New Zealand, 25–45 m depth), and one large *Bathymodiolus* at 154 m (Makauley Cone, Kermadec Ridge) (Smith et al. 2004; Dando 2010). Vent- and seep-specialist chemosymbiotic metazoans are more significant in the transition zone between the shelf and the deep sea. In the Gulf of Mexico, 37% of the species identified at various seep sites ranging from 400 to 1000 m on the Louisiana slope harbored symbionts (Sibuet and Olu 1998). At these depths, symbiotic nutrition can be mixed with assimilation of photosynthesis-derived carbon, as demonstrated in the symbiont-bearing mussel *Bathymodiolus azoricus* from the Menez Gwen site (800 m deep on the Mid-Atlantic Ridge), which obtain part of their nutrition from heterotrophy, and not only from their symbionts (Riou et al. 2010). Besides, many groups of deep-sea chemosymbiotic organisms are supposedly derived from shallow-water ancestors (Distel et al. 2000). Sites situated in the transition zone between shelf and deep sea are thus of particular interest to understand the relative importance of symbiotic versus heterotrophic nutrition, and how seep- and vent-specialist faunas with their bacterial partners have evolved.

Research on cold seeps in the Gulf of Guinea is linked to the existence of important hydrocarbon reservoirs. Initial studies focused on geophysical aspects, followed by a more biology-oriented program which benefited from several cruises. Along with taxonomic identification of organisms (Andersen et al. 2004; von Cosel and Olu 2009), a major concern was the assessment of deep-sea drilling impact (Jorissen et al. 2009). Most of the work focused on the deep site Regab, a giant pockmark located 3150 m deep on the Gabon margin, due to its biological richness (Olu-LeRoy et al. 2007a,b; Cambon-Bonavita et al. 2009; von Cosel and Olu 2009). Yet, only a few molecular studies investigated organisms and their associated symbionts at Regab (Duperron et al. 2005; Cambon-Bonavita et al. 2009; Rodrigues and Duperron 2011). The shallower Guiness site was discovered 490 km north of Regab, at depths between 580 and 670 m, but was not explored in detail (von Cosel and Olu 2009). Fauna at Guiness is composed mostly of vagrant metazoans such as crabs, holothurians, anemones, and various fishes, which likely rely on photosynthesis-derived organic matter. Seep specialists such as vesicomyid clams and siboglinid tubeworms are also present, in lower abundances, associated with carbonates and localized patches of dark sediment often covered by white microbial mats (Fig. S1F and H, pers. obs.). Large numbers of empty bivalve shells are seen. During the 2011 WACS (“West African Cold Seeps”) cruise, several metazoans from groups known to live in symbiosis with bacteria were sampled at Guiness, for which no data were previously available. Faunal similarities exist between Gulf of Guinea and Gulf of Mexico cold seeps, and it is hypothesized that a colonization route exists along the Atlantic Equatorial Belt. Documenting organisms and symbioses in the Gulf of Guinea at several depths is thus of major interest if we are to understand the biogeography of chemosynthetic symbioses (Olu-LeRoy et al. 2007a,b; Olu et al. 2010). In this study, we investigated bacterial symbioses in five metazoan species, four bivalve molluscs: *Acharax* sp. (Solemyidae), *Thyasira* sp. (Thyasiridae), *Elenaconcha guiness*, and *Calyptogena valdiviae* (Vesicomyidae), and one annelid species, *Lamellibrachia* sp. (Siboglinidae). Hosts and associated bacteria were characterized by marker gene sequencing (28S rRNA for metazoans, 16S and 23S rRNAs for bacteria). Occurrence of bacteria in animal tissue was confirmed using fluorescence in situ hybridization (FISH) on metazoan tissue sections. Presence of diagnostic genes encoding particular bacterial metabolic functions, namely adenosine 5′-phosphosulfate (APS) reductase involved in sulfur metabolism and ribulose-1,5-bisphosphate carboxylase oxygenase (RubisCO) involved in autotrophic carbon fixation through the Calvin cycle, was tested using specific polymerase chain reaction (PCR) primer sets. Nutrition strategies were investigated using stable isotope analyses (^13^C, ^15^N, and ^34^S) of bacteria-containing tissues. Data from this study were the first available regarding symbiotic associations at the Guiness site in the Gulf of Guinea, a region which is only beginning to be investigated. Results will help documenting and understanding large-scale diversity, biogeographical and depth-related patterns of chemosynthetic symbioses.

## Material and Methods

### Specimen sampling

Specimens were collected at the Guiness Site (Gulf of Guinea) during the WACS cruise aboard RV *Pourquoi Pas?* using the ROV *Victor 6000* in February 2011 (ROV dive 433, 1°34.65′S, 8°32.91′E, 582 m depth). During the dive, faunal sampling yielded a variety of metazoans, among which those belonging to groups reported as symbiotic were investigated here (Fig. S1). These included representatives of four bivalve species investigated here, namely *Acharax* sp. (Solemyidae), *Thyasira* sp. (Thyasiridae), *E. guiness*, and *C. valdiviae* (Vesicomyidae). *Isorropodon* sp. was also collected and is presented in a separate study (Rodrigues et al. 2012). The annelid species, *Lamellibrachia* sp. (Siboglinidae) was also studied. *Acharax* sp. and juvenile *E. guiness* were recovered within deeper dark sediment in the blade core CL10 in bare, brown sediment next to vesicomyid aggregates. *Calyptogena valdiviae* was sampled using a net sampling (Net 2, Fig. S1F) within a patch of vesicomyids. *Thyasira* specimens were recovered using a net sampling (Net3, Fig. S1H) operated on reduced sediment where empty shells were seen on the bottom. *Lamellibrachia* sp. specimens were collected from a “tubeworm bush” popping up from a crack between two large carbonates. Because of that, only the anterior part could be sampled using the ROV grab, including plume, vestimentum, and part of the trunk, but not the opisthosome. For each of these species, three (labeled 1–3) or more specimens were dissected and appropriate tissues, that is, gills for bivalves and trunk (posterior to the vestimentum) for the siboglinid, were sampled. Tissues were frozen for stable isotope analyses, fixed in ethanol for molecular characterization, fixed in 4% formaldehyde (4°C, 2–4 h).

### Molecular characterization

DNA was extracted from tissue fragments of three specimens per species using the DNA Blood and Tissue Kit™ (Qiagen, CA). Fragments of metazoan 28S rRNA-encoding genes were PCR amplified and products sequenced. Bacterial genes encoding 16S rRNA were amplified, three separate PCR products were generated from each specimen and pooled, then purified using a QiaQuick Kit™ (Qiagen), and cloned using a TOPO TA™ Cloning Kit (Invitrogen, CA). Inserts were sequenced in both directions. Fragments of bacterial genes, some diagnostic of metabolic capabilities, were tested using various primer sets. These included genes encoding 23S rRNA, APS reductase, RubisCO form II. PCR products were sequenced directly, and if sequence displayed no ambiguity such as double peaks, no additional cloning step was added. Other genes were tested, but yielded no product, namely RubisCO form I, and particulate methane mono-oxygenase (pmoA, the enzyme responsible for aerobic methane oxidation). All primers and PCR programs used are summarized in Table 1. Primers for pmoA did not amplify any product in any specimen, only positive controls did produce a band. All products were sequenced by GATC Biotech (Constanz, Germany), and are registered at EMBL under accession numbers HE863781–HE863806.

**Table 1:** Primers and PCR parameters used for amplifications

Gene	Annealing (cycles)	Primer name	Primer sequence (5′→3′)	References
16S	45°C (27)	27F	AGAGTTTGATCATGGCTCAG	Lane (1991)
		1492R	GTTACCTTGTTACGACTT	
23S	53°C (35)	3505F	GACCGTCAGCTAAGGTCCCAA	Stewart et al. (2009)
		4761R	CCAGTCAAACTACCCACCATG	
APS reductase	58°C (25)	APS1-FW	TGGCAGATCATGATYMAYGG	Meyer and Kuever (2007)
		APS4-RV	GCGCCAACYGGRCCRTA	
RuBisCO II	62°C (25)	cbbm1_Els	ATCATCAARCCSAARCTSGGCCTGCGTCC	Blazejak et al. (2006)
		cbbm2_Els	MGAGGTGACSGCRCCGTCRCCRGCMCGRTG	
Pmoa	55°C (30)	A189F	GGNGACTGGGACTTCTGG	Holmes et al. (1995)
		M661R	CCGGMGCAACGTCYTTACC	
28S	52°C (35)	28S-C1	ACCCGCTGAATTTAAGCAT	Williams et al. (2004)
		28S-C2	TGAACTCTCTCTTCAAAGTTCTTTTC	
RubisCO I	55°C (35)	cbbl_1b	CACCTGGACCACVGTBTGG	Blazejak et al. (2006)
		cbbl_2c	CGGTGYATGTGCAGCAGCATNCCG	

### Analysis of gene sequences

Sequences were compared with sequences available in Genbank using BLAST (Altschul et al. 1990). Nucleic acid sequences of bacterial genes encoding ribosomal RNAs were analyzed, while amino acid sequences were used for protein-encoding genes. Data sets were built for each gene including BLAST hits, symbiont, and reference sequences. Alignments were performed using SINA Web aligner (16S rRNA) or ClustalW (other genes), manually checked and truncated (Pruesse et al. 2007). Phylogenetic analyses were performed using Maximum Likelihood approaches with randomization of sequence input order using the PHYLIP package under a GTR model for nucleic acid sequences and a Jones–Taylor–Thornton model for amino acid sequences (Felsenstein 2002), and using Bayesian approaches with a GTR model and γ rates with a proportion of invariants (DNA sequences) and a mixed model of amino acid substitution (amino acid sequences) run for 200,000–500,000 generations using Mr. Bayes 3.2.1 (Ronquist and Huelsenbeck 2003). Bootstraps values were obtained from 500 or 1000 replicates analyzed using F84 distances and Neighbor-Joining.

### Symbiont localization in metazoan tissue

Fragments of gill (bivalves) and trunk (*Lamellibrachia* sp.) fixed for FISH were dehydrated in increasing ethanol series, embedded in polyethylene glycol distearate : 1-hexadecanol (9:1), cut, and hybridized for 1–3 h at 46°C as previously described (Duperron et al. 2008). Probes and formamide concentrations used are summarized in Table 2. A new probe, Creg821 (5′-GTACCCCCCCCAACGACT-3′), was designed to target exclusively the symbionts of *C. valdiviae* and was tested against gill tissue of *Laubiericoncha chuni*, a species from the Gulf of Guinea of which the symbiont displays a one-base mismatch in the middle of the sequence. It was shown to hybridize specifically at 30% formamide. All probes were tested with both Cy-3 and Cy-5 fluorochromes. Hybridized sections were visualized under an Olympus BX61 epifluorescence microscope (Olympus, Japan).

**Table 2:** FISH probes used in this study, with position in the *Escherichia coli* 16S rRNA sequence, percentage of formamide in hybridization buffer, and target groups

Probe	Sequence (5′→3′)	Position	% Formamide	Target	Reference
EUB338	GCTGCCTCCCGTAGGAGT	338	20–40	Most eubacteria	Amann et al. (1990)
GAM42	GCCTTCCCACATCGTTT	42	30	γ-proteobacteria	Manz et al. (1992)
EPSY549	CAGTGATTCCGAGTAACG	549	50	ε-proteobacteria	Manz et al. (1992)
CF319	TGGTCCGTGTCTCAGTAC	319	40	Bacteroidetes	Manz et al. (1996)
LaSp60	CCATCGTTACCGTTCGAC	60	40	Cohybridization with LaSp640: vestimentiferan symbionts	Duperron et al. (2009)
LaSp640	CACACTCTAGTCAGGCA	640	40	Cohybridization with LaSp60: vestimentiferan symbionts	Duperron et al. (2009)
ImedT2	TAGAGGCCTCCTTTA	193	30	Mytilid thiotrophic symbionts	Duperron et al. (2008)
ThyGui138	TTCCACAGGTTGTCC	138	40	If positive and ThyGui642 negative: thyasirid Guiness symbiont	Rodrigues and Duperron (2011)
ThyGui642	TCTAGTTGAACAGTT	642	40	If negative and ThyGui138 positive: thyasirid Guiness symbiont	Rodrigues and Duperron (2011)
Creg821	GTACCCCCCCCAACGACT	821	30	Thiotrophic symbiont of *Calyptogena valdiviae* specimen 1	This study

### Stable isotope analyses

Fragments of frozen tissue (gill for bivalves, trunk for *Lamellibrachia*) were dried (4 days, 60°C) and ground to powder using mortar and pestle. To avoid biases in nitrogen measurements, no HCl treatment was applied (Kaehler and Pakhomov 2001). Values of δ^13^C and δ^15^N were determined and are expressed as relative per-mL (‰) differences between samples and Pee Dee Belemnite (PDB) and air N_2._ Values for sulfur could only be measured in *Lamellibrachia* and *C. valdiviae*, and δ^34^S was calculated against Canyon Diablo Troilite. Analyses were performed using a GV Isoprime (U.K.) Stable isotope mass spectrometer using reference standards IA-R042, IA-R045, IA-R046, IA-R005 IA-R006 (nitrogen and carbon) and IA-R027, IA-R061, and IAEA SO5 (sulfur) at Iso-Analytical (Crewe, U.K.).

## Results

### Host 28S rRNA gene sequences

Some metazoans collected from the Guiness site are described by taxonomists, but molecular data are not yet available and is thus included here. Because cryptic species are reported in the groups here investigated, a molecular marker was used to address whether all specimens investigated in each group belong to a single species, and to provide reference for comparison with future work. Fragments of 28S rRNA were sequenced in order to check whether all specimens or a given morphospecies actually represented a single species, and all specimens within a single morphospecies yielded identical sequences. The *Acharax* sp. sequence displayed 91% similarity with that of *Solemya togata* and *S. velum*. The thyasirid specimens harbored a sequence strictly identical to sequences reported in *Thyasira sarsi* from North Sea and *T. methanophila* from near Concepciòn, Chile. It thus likely belongs to the genus *Thyasira* and will be referred to as *Thyasira* sp. Guiness below. The *E. guiness* and *C. valdiviae* sequences differed by 1% and were most similar (99%) to the sequence from *C. pacifica*, although it must be noted that few 28S rRNA sequences are available from vesicomyids. *Lamellibrachia* sp. 28S rRNA was over 99% identical with that of various siboglinid annelids including *Riftia pachyptila*, *Sclerolinum brattstromi*, *Ridgeia* sp., and the aplacophoran *Chaetoderma* sp. (the latter sequence could be questioned, as it is the only nonannelid sequence in over 100 blast hits). It displayed 98% identical positions with the congeneric *L. satsumae*.

### Bacterial 16S and 23S rRNAs

**Figure 1:**
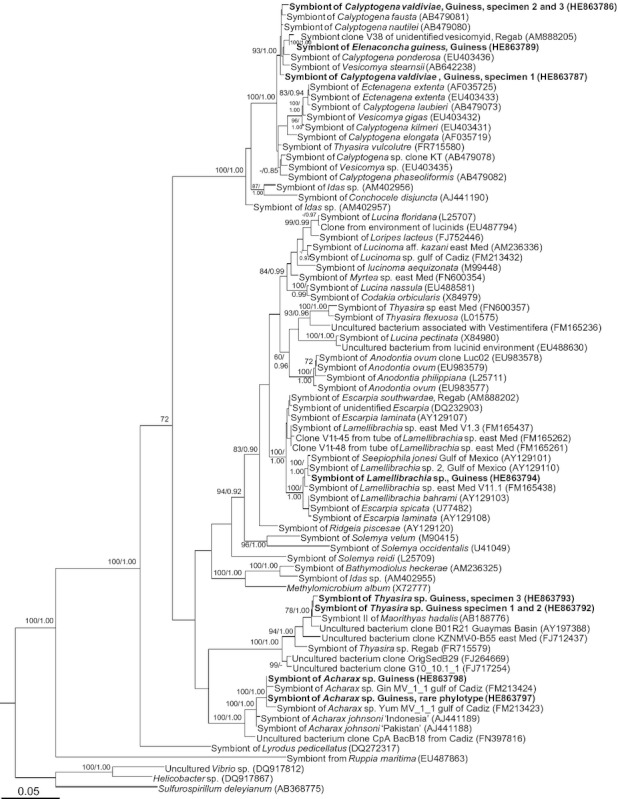
Phylogenetic tree based on the analysis of the bacterial 16 rRNA-encoding gene (1315 nucleotide positions analyzed). Sequences from this study are in bold. For methodology, see Material and Methods. Bootstraps calculated from 1000 NJ replicates (>60 shown) and Bayesian posterior probabilities (>0.85 shown) are displayed at nodes as “bootstrap/probability.” Scale bar represents 5% estimated sequence divergence.

All three *Acharax* specimens displayed the same dominant 16S rRNA phylotype (77/80 clones). A second phylotype, differing by only three of 1504 nucleotides, was detected in a single specimen (three clones). Both phylotypes displayed 99% identical positions with the endosymbiont of *Acharax johnsoni* from Pakistan seeps. They clustered with several symbionts from *Acharax* species and with a γ-proteobacterial sequence from the Captain Arutyunov Mud Volcano (MV) in the Gulf of Cadiz (Fig. 1). *Thyasira* sp. Guiness sequence from specimen T3 differed slightly (6/1499 nt) from that found in the two other specimens, and both displayed 99% similarity with a shorter sequence from the symbiont of the thyasirid *Maorithyas hadalis*. Other close relatives (98% identity) included the symbiont of *Thyasira* sp. from Regab, and sequences from the Guaymas Basin vents (Pacific) and Kazan Mud Volcano (Mediterranean). All clustered together in the tree (Fig. 1). *Elenaconcha guiness* displayed a single 16S rRNA phylotype. *Calyptogena valdiviae* specimen 1 displayed a single 16S rRNA sequence (24 clones) differing by 1% from that found in specimens 2 and 3 (24 and 20 clones, respectively). This sequence also occurred once in specimen 2. *Elenaconcha guiness* and *C. valdiviae* sequences were similar to that of various symbionts associated with vesicomyid clams (98–99% identity) and clustered within the same group of vesicomyid-associated symbionts (Fig. 1). The *E. guiness* sequence was most closely related to a sequence from an undetermined vesicomyid clam collected at the Regab site. The 16S rRNA phylotype associated with *Lamellibrachia* sp. was highly similar to phylotypes recovered from siboglinids within genera *Lamellibrachia*, *Escarpia,* and *Seepiophila* (98–99% similarity) from sites in the Pacific Ocean, Gulf of Mexico, and Mediterranean, and all clustered together in the tree (Fig. 1).

**Figure 2:**
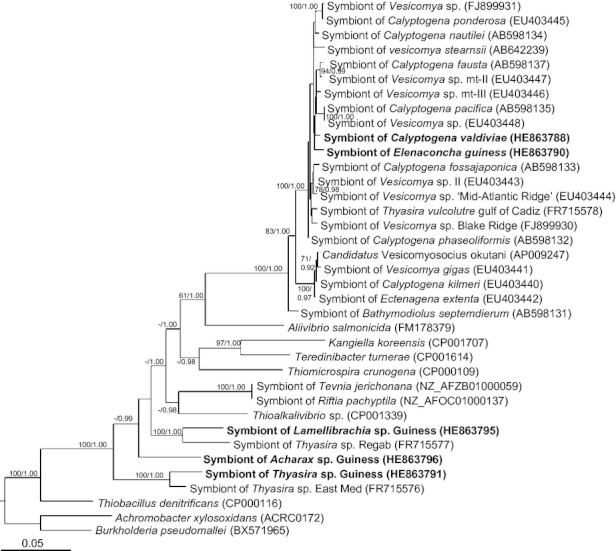
Phylogenetic tree based on the analysis of the bacterial 23 rRNA-encoding gene (1153 nucleotide positions analyzed). Sequences from this study are in bold. For methodology, see Material and Methods. Bootstraps calculated from 1000 NJ replicates (>60 shown) and Bayesian posterior probabilities (>0.85 shown) are displayed at nodes as “bootstrap/probability.” Scale bar represents 5% estimated sequence divergence.

Within a given host species, all specimens displayed identical 23S rRNA-encoding gene fragment sequences. The sequence from *Acharax* sp. was most similar to that of *Thioalkalovibrio sulfidophilus,* but with only 88% identical positions, and branched between two groups including other sequences from this study (Fig. 2). The first group included the sequence from *Thyasira* sp. Guiness and its close relative, the sequence from another *Thyasira* from the eastern Mediterranean (95% identical positions). The second group included the *Lamellibrachia* sp. sequence from this study and its closest relative, the sequence from *Thyasira* sp. from Regab. The 23S rRNA sequence obtained from *E. guiness* was highly similar (>98%) to several vesicomyid sequences with which it clustered in the tree (Fig. 2). For *C. valdiviae*, in which two 16S rRNA sequences were identified, 23S rRNA was cloned and 94 clones analyzed (∼31 per specimen). All sequences were identical, and 99% similar to those of *C. fausta*, *C. nautilei*, and *C. phaseoliformis*.

### APS reductase and RubisCO

**Figure 3:**
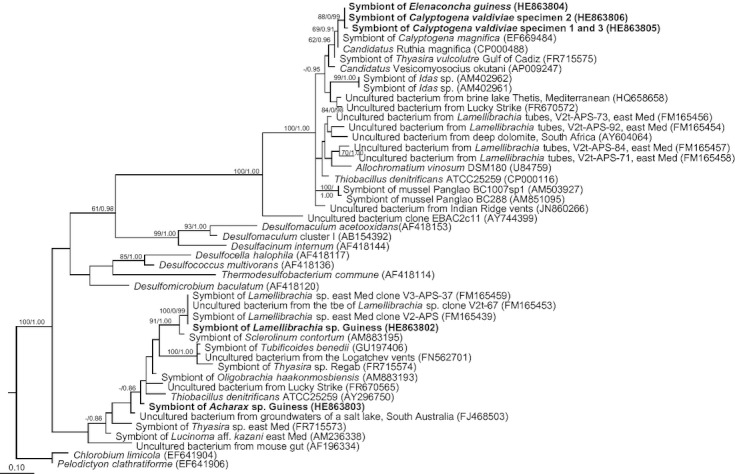
Phylogenetic tree based on the analysis of a fragment of the APS reductase-encoding gene (127 amino acid positions analyzed). Sequences from this study are in bold. For methodology see Material and Methods. Bootstraps calculated from 500 NJ replicates (>60 shown) and Bayesian posterior probabilities (>0.85 shown) are displayed at nodes as “bootstrap/probability.” Scale bar represents 10% estimated sequence divergence.

Fragments of APS-encoding gene sequences were obtained from all specimens except for the three thyasirids, despite several attempts using various PCR conditions for the latter. Within a given host species, all three specimens displayed identical sequences except for *C. valdiviae* for which a cloning step was necessary. The APS sequence from *Acharax* sp. displayed 95% identical amino acid positions with the sequence from the Arctic Ocean siboglinid *Oligobrachia haakonmosbiensis*, and with a sequence from ground waters in an evaporative lake. The sequence branched between the two mentioned above, along with *Thiobacillus denitrificans* and an environmental sequence from the Lucky Strike vent field, but with low bootstrap support (Fig. 3). The APS sequence from *E. guiness* was highly similar to several sequences from vesicomyid symbionts, including “*Cand*. Vesicomyosocius okutanii” and *Ruthia magnifica* (>98% identical aa positions). *Calyptogena valdiviae* specimens 1 and 3 displayed the same APS nucleic acid sequence, differing from that of specimen at 2–4% positions (43 clones analyzed). These were mostly silent substitutions, as a single amino acid out of 120 was different. APS nucleic acid sequences were 2–4% different from that of *E. guiness*, representing 0–1 amino acid. *Calyptogena valdiviae* and *E. guiness* sequences clustered together with good support (bootstrap value of 88, posterior probability of 0.99) within the clade including vesicomyid sequences and a sequence from *Thyasira vulcolutre* (Fig. 3). The amino acid sequence from the siboglinid *Lamellibrachia* sp. was identical to one of the two sequences documented from a congeneric species in the Eastern Mediterranean, and branched within a clade of siboglinid APS sequences (Fig. 3).

RubisCO form II was successfully amplified only from *Acharax* sp. and *Lamellibrachia* sp. In *Acharax* sp., the nucleic acid sequence from specimen 2 displayed five differences over 335 positions with the two other specimens (A1 and A3). Because these differences were spread over the whole length of the sequence, and no double peaks were seen in the chromatogram, we suggest this reflects a real difference. They were, however, all synonymous, and the amino acid sequences from all three specimens were strictly identical. Among best BLAST hits, the sequence was 93% identical with that from two siboglinids, *Lamellibrachia anaximandri* from the eastern Mediterranean, and *Sclerolinum contortum* from the Arctic Ocean, but also with several environmental sequences from oil-loaded sediment and suboxic habitats. In the tree, the sequence appeared close to that of *S. contortum* and some environmental sequences (Fig. S2). All three specimens of *Lamellibrachia* sp. displayed identical nucleic acid sequences, and amino acid sequence was most similar to sequences from a *Lamellibrachia* sp. from Sagami Trough near Japan (98%), *Tubificoides benedii* (95%), two environmental sequences from a sulfide chimney in the Suiyo Seamount (98%), and one from the Eiffel Tower on the Mid Atlantic Ridge (94%). It clustered with the *Lamellibrachia* sequence in the tree, but surprisingly away from sequences from the eastern Mediterranean species (Fig. S2). Despite repeated attempts, no RubisCO form II was obtained from *E. guiness*, *C. valdiviae*, and *Thyasira* sp. Guiness. No RubisCO form I sequence was obtained from any of the specimens using primer sets employed.

### Fluorescence in situ hybridization

**Figure 4:**
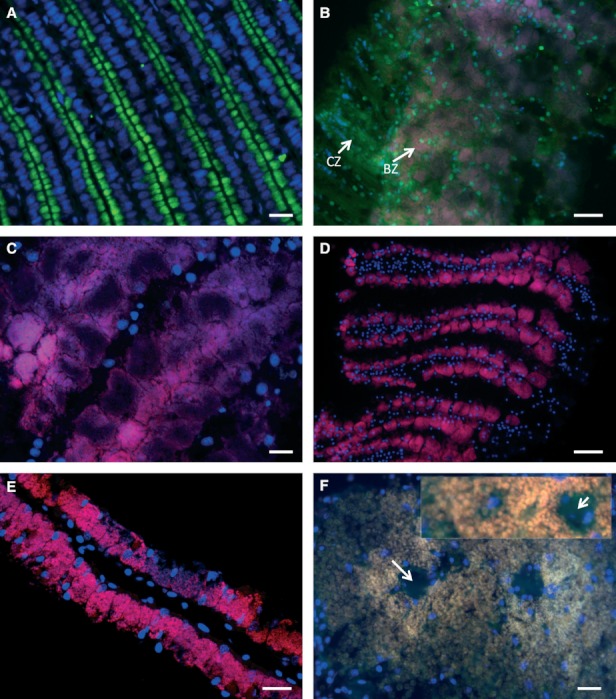
Fluorescence in situ hybridization on cross-sections of metazoan tissue. All slides were counterstained with DAPI (host nuclei visible in blue). Scale bars = 20 μm (A, C, E, F) and 50 μm (B, D). (A) Gill filaments of *Acharax* sp. labeled with probe Gam42 in green (Cy-5). (B) Gills of *Thyasira* sp. Guiness labeled with probes ThyGui642 (Cy-3, in green) and ThyGui138 (Cy-5, in red, yielding pink with addition of DAPI). Ciliated zone (CZ) is mostly devoid of bacteria, appearing green because of autofluorescence. The bacterial signal corresponds to the pink area, located on the lateral zone (bacteriocyte zone BZ) of the gill filament. (C) Another view of the densely colonized lateral zone of *Thyasira* gill filament. Bacteria (probe Gam42) appear in pink. (D) Gill filaments of *Elenaconcha guiness* with ImedT2 signal in red. (E) Gill filament of *Calyptogena valdiviae* specimen 1 hybridized with probe Creg821 (Cy-5, pink). (F) Cross-section through the trunk of *Lamellibrachia* sp. Individual lobes are visible, each organized around a central lumen (arrow on image and upper right insert). Two lobes are visible. Notice the smaller and brighter bacteria closer to the central lumen (image and insert). Signals from probes LaSp640 (Cy-3, in green) and LaSp60 (Cy-5, in red) fully overlap, yielding a yellow color.

The presence of bacteria was investigated in the gill tissues of bivalve specimens. *Acharax* sp. gills appeared as typical protobranch ctenidia, and displayed abundant filaments composed of two layers of epithelial cells separated by a space where hemolymph circulates. Bacteria were located in the lateral zone of gill filaments, and occurred in very dense populations in the apical half of epithelial cells, while nuclei were basal (Fig. 4A). All bacteria hybridized with the Gam42 probe and signal fully overlaid that of probe Eub338.

Gill tissue of *Thyasira* sp. Guiness was composed of filaments which harbored bacterial symbionts only in part of the lateral zone (Fig. 4B). As in several other thyasirids, bacteriocytes appeared as bags filled with bacteria, with very little to no cytoplasm, and bacteria reached very high densities (Fig. 4C). Nuclei were sometimes located in the apical part of bacteriocytes, but this might be an artifact resulting from fixation. Two probes, ThyGui138 and ThyGui642, both designed to hybridize symbionts of *Thyasira* sp. from Regab, were used (Rodrigues and Duperron 2011). ThyGui138 had no mismatch with the identified 16S rRNA phylotypes in *Thyasira* sp. Guiness, while ThyGui642 had one mismatch in the middle of the target region. As expected, all bacteria hybridized with the former and not the latter at 40% formamide (a faint signal was seen with ThyGui642 at 30% formamide). This strongly suggests that the identified 16S rRNA phylotypes corresponded to the dominant symbionts. Probes Gam42 and Eub338 gave signals, while Cf319 and Epsy549 did not.

Bacteria were abundant in the lateral zone of gill filaments in *E. guiness* and *C. valdiviae*, and hybridized successfully with Eub338. Specimen of *E. guiness* hybridized successfully with ImedT2, initially designed to target mussel sulfur-oxidizing symbionts, but targeting several vesicomyid symbionts, which are closely related (Fig. 4D). Symbiont of *C. valdiviae* specimen 1 hybridized successfully with Creg821, a probe designed to target the 16S rRNA phylotype identified in specimen 1, and displaying two mismatches with the phylotype recovered in the two other specimens (Fig. 4E). Specimens 2 and 4 did not display any hybridization using probe Creg821. Probe Gam42 did not yield a signal, indicating that it might not target the 23S sequence in *E. guiness* and *C. valdiviae*, but this is not possible to check because the target region of the probe was not part of the 23S rRNA fragment sequenced here. As in other bivalves observed in this study, bacteria occurred in dense populations in gill epithelial cells of the lateral zone of gill filaments.

In *Lamellibrachia* sp., bacteria were detected from the anterior to the posterior part of the trunk, posterior to the vestimentum. They occupied most of the volume within the trophosome, and all hybridized with probes Eub338, Gam42, and with probes LaSp60 and LaSp640, which were initially designed in a way that dual hybridization would be specific for symbionts of a *Lamellibrachia* species from the eastern Mediterranean (Fig. 4F, Duperron et al. 2009). These symbionts were highly similar to the 16S rRNA phylotype recovered here, and no mismatch occurred between our sequence and both probe sequences, supporting that they actually represented the dominant symbiont. Bacteria of various sizes occurred, some of which possessed sulfur granules recognizable because of their typical greenish/yellowish coloration when exposing crushed fresh tissue to transmitted light. The trophosome was organized in lobes, and bacteria surrounded a central canal, as evident on some sections. Smallest bacteria occurred immediately around the canal, and bacteria appeared larger as one looked away from the center (Fig. 4F illustrates two such lobes). Around lobes, many host nuclei could be seen and bacteria were no longer visible.

### Stable isotope analyses

Ratios of dry weight percentage of carbon to nitrogen were in the range of expected values for metazoans, that is, between 3 and 4, and are summarized, along with signatures, in Table 3. *Acharax* sp. gills had to be pooled to obtain enough material, and yielded δ^13^C and δ^15^N values of −-29.7‰, and 1.5‰, respectively. *Thyasira* sp. Guiness yielded mean values of −35.8 ± 0.8 and 5.6 ± 1.2‰; *E. guiness* and *C. valdiviae* had mean values of −35.9 ± 0.2 and 1.4 ± 0.1‰ and −35.8 ± 0.8 and −1.0 ± 2.2‰, respectively. Finally, *Lamellibrachia* sp. displayed mean values of −30.9 ± 2.6 and 2.0 ± 1.6‰. Sulfur stable isotope signature could only be measured in specimens of *Lamellibrachia* and *C. valdiviae*. In the siboglinid, sulfur represented 2.2–2.6% of the biomass, with mean δ^34^S values of −23.4 ± 2.0‰. In *C. valdiviae*, mean δ^34^S values of 5.9 ± 1.3‰ were obtained.

**Table 3:** Percentage in tissue dry weight and stable isotope signatures (in ‰ against standards) of nitrogen, carbon, and sulfur

Species	Specimen ID	% N	δ^15^N	% C	δ^13^C	% S	δ^34^S
*Acharax* sp.	A2 + A3	10.4	1.5	41.6	−29.7		
*Thyasira* sp.	T1	10.0	7.0	37.0	−36.6		
	T2	9.3	6.1	37.7	−36.4		
	T3	9.7	4.3	37.7	−35.1		
	T4	10.1	5.1	38.4	−35.2		
*Elenaconcha guiness*	V1	10.2	1.4	37.6	−36.1		
	V2	9.6	1.3	35.0	−35.8		
*Calyptogena valdiviae*	V1	9.6	−3.5	40.3	−34.7	5.3	5.5
	V2	8.2	0.8	36.7	−36.3	4.4	7.4
	V4	9.4	−0.3	40.2	−36.4	5.3	4.8
*Lamellibrachia* sp.	S1	6.9	0.4	40.3	−30.1	2.2	−25.6
	S2	9.6	2.0	43.8	−33.8	2.3	−23.1
	S3	10.4	3.5	43.8	−28.7	2.6	−21.6

A2 + A3 indicates that samples from specimens A2 and A3 needed to be pooled so that enough material was available.

## Discussion

### Evidence for sulfur-oxidizing symbioses in metazoans from Guiness

The data presented here support that five species were investigated based on 28S rRNA gene analysis, a gene slightly more variable than 18S rRNA in bivalves, recently employed to improve species characterization in several families (Taylor et al. 2007). Each species harbors a single dominant bacterial symbiont type located in the gill tissue for bivalves (*Acharax* sp., *Thyasira* sp., *E. guiness*, and *C. valdiviae*), and in the trophosome for the siboglinid *Lamellibrachia* sp. Symbiont densities observed by FISH are high in all cases, supporting the hypothesis of a major contribution of bacteria to their host's physiology and nutrition. All symbionts are closely related to sulfur-oxidizing bacterial symbionts and to environmental bacteria from sulfur-enriched deep-sea reducing environments based on 16S and 23S rRNA sequence analyses. Although 16S rRNA is sufficient for basic characterization, the 23S rRNA-encoding gene can be a target for FISH probes and is also a valuable phylogenetic marker. Database for the latter does not contain many sequences at this stage, but this gene should be considered, in combination with 16S rRNA, for future studies of symbiont biogeography and evolution. Putative sulfur-linked metabolism of symbionts is supported by the presence of an APS reductase-encoding gene in all cases except *Thyasira* sp. Guiness. In this latter species, the symbiont is, however, related to the symbiont of *Thyasira* sp. Regab from which an APS sequence was recently obtained (Rodrigues and Duperron 2011). It is thus possible that the lack of an APS sequence here is due to mismatches in the primers' target regions rather than due to absence of a sulfur metabolism. Autotrophy can be postulated for symbionts of *Acharax* sp. and *Lamellibrachia* sp. thanks to the presence of Type II RubisCO. This type of RubisCO is expected in Siboglinidae, but not in *Acharax*, as symbionts of another Solemyidae, *Solemya velum*, were reported to harbor Type IA RubisCO (Schwedock et al. 2004; Stewart et al. 2005). Type II RubisCO is also reported in Vesicomyidae, it is thus surprising that it could not be obtained here.

Carbon stable isotope signatures measured here range from −28.7 to −36.6‰, within the range of values expected for chemoautotrophy-derived carbon (−25 to −40‰) (Fisher 1995). Values for δ^13^C and δ^15^N in *Acharax* sp. Guiness are comparable with values reported for other deep-sea solemyids (−28 to −35‰ for carbon, −3.4 to 6.1‰ for nitrogen for seep species in the Gulf of Cadiz (Rodrigues et al. 2010)). In *S. velum*, similar carbon signatures yielded an estimated bacterial contribution to host carbon up to 98% (Conway et al. 1989). In thyasirids, values between −37.2 and −29.0‰ for carbon, and −5.2 and 4.8‰ for nitrogen are reported from seep species in the Gulf of Cadiz and eastern Mediterranean (Carlier et al. 2010; Rodrigues et al. 2010). Carbon values reported here are within this range, while nitrogen values are slightly higher (from 4.3 to 7.0‰). Values for *E. guiness* and *C. valdiviae* are within the range of values commonly reported for seep vesicomyids, including those from the nearby Regab site (δ^13^C around −36.0‰, δ^15^N between −1.0 and 3.5‰; Olu et al. 2009). Values reported for *Lamellibrachia* sp. from Guiness are close to values obtained from species collected at sites such as Regab or the eastern Mediterranean (δ^13^C between −36.2 and −24.2‰ and δ^15^N between 1.9 and 2.5‰; Duperron et al. 2009; Olu et al. 2009. Thanks to the symbiont's ability to store sulfur granules in Siboglinidae, δ^34^S could be measured and yielded highly negative values similar to those of various organisms with sulfur-oxidizing symbionts (Fry et al. 1983; Vetter and Fry 1998; Carlier et al. 2010). Overall, δ^13^C values measured in all five species are in the range of values reported from other species within their families for which a sulfur-oxidizing symbiosis is documented. It supports that *Acharax* sp., *E. guiness*, *C. valdiviae,* and *Lamellibrachia* sp. harbor sulfur-oxidizing symbioses and derive most of their carbon from bacterial chemoautotrophy. *Thyasira* sp. Guiness also likely harbors a sulfur-oxidizing symbiosis, but our inability to amplify APS reductase makes the conclusion less affirmative. Its less-negative nitrogen signature compared with other species also suggests that it uses distinct nitrogen sources.

### Comparison with other symbioses

*Acharax* sp. Guiness harbors symbionts which are closely related to those of *Acharax* species from the Gulf of Cadiz (Yuma and Ginsburg MVs, around 900 m depth), but also from very distant locations including seeps near the Oregon, Pakistan, or Indonesia and various depths from 780 to 2940 m (Imhoff et al. 2003; Rodrigues et al. 2010). They all form a tight cluster suggesting monophyly of *Acharax*-associated symbionts. Although hosts belong to the same family, *Acharax* symbionts are not closely related to symbionts of *Solemya*, suggesting that differences exist between these two symbioses. For example, the maternal inheritance of symbionts documented in *Solemya* cannot be inferred for *Acharax* without a dedicated study (Cary 1994). Similarly, *Acharax* symbionts appear to possess a Type II RubisCO not closely related to the Type IA RubisCO identified in *Solemya velum* (Schwedock et al. 2004). The Solemyidae family dates back to at least the middle Ordovician, 460–480 MYA (Pojeta 1988), and symbiosis was supposed to have been established prior to the diversification (Distel 1998). Symbionts should thus be related, but it is also possible that symbionts were replaced by new bacteria during evolution of the group, or that differences arose because of recombination events involving metabolic pathways in bacteria. This results in the lack of host–symbiont cospeciation patterns. The distinct symbionts in *Acharax* and *Solemya* species might be linked to their distinct depth distribution, as *Acharax* are usually deeper-dwelling species than *Solemya*. Data regarding deep-sea Solemyidae symbioses are needed before these hypotheses can be properly tested, as most studies investigated shallower species *Solemya velum* and *S. reidi* (Stewart and Cavanaugh 2006).

For all three bacterial genes investigated, *E. guiness* and *C. valdiviae* specimens displayed sequences with high similarity to available vesicomyid-associated symbiont sequences. This indicates that they display similar type of symbiosis. Unexpectedly, specimen 1 of *C. valdiviae* displayed a 16S rRNA symbiont phylotype distinct from that of the two other specimens, a result confirmed by FISH. Sequences from the 23S rRNA were identical, meanwhile APS sequence from specimen 2 was different from that of specimen 1 and 3. This reveals a certain level of genetic heterogeneity in this species, and may illustrate intraspecific variation in dominant symbiont strain. This is in line with recent work evidencing occasional nonmaternal inheritance of symbionts in Vesicomyidae (Stewart et al. 2008). At Guiness and at the nearby site Regab, several vesicomyid species are documented, some co-occurring in the same aggregates (von Cosel and Olu 2009). Future work will thus test whether co-occurrence could favor symbiont gene or strain exchanges.

Gill structure of *Thyasira* sp. Guiness corresponds to the Type 3 described in *Thyasira* and *Conchocele* by Dufour (Fig. 1D in Dufour 2005). Patterns observed in FISH images resemble those obtained for other *Thyasira* species from the eastern Mediterranean, Gulf of Cadiz and Regab (Brissac et al. 2011; Rodrigues and Duperron 2011). Thyasirid-associated symbionts belong to at least three distinct lineages within γ-proteobacteria, suggesting multiple independent events of symbiont acquisition during evolution (Imhoff et al. 2003; Rodrigues and Duperron 2011). Symbiont 16S rRNA sequences from *Thyasira* sp. Guiness clusters with symbionts of *M. hadalis* (Fujiwara et al. 2001) in a group, which also includes symbionts from *Thyasira* sp. Regab. Specimen 3 displayed a slightly different 16S (but identical 23S) rRNA sequence compared with the two other conspecifics, which could indicate uptake of a slightly distinct bacterial strain. It must be noted that contrary to the 16S, the 23S rRNA sequence clusters with the *Thyasira* sp. from the eastern Mediterranean, and not with that of *Thyasira* sp. Regab. These last two points indicate that strain heterogeneity within and among members of a single thyasirid species should be further investigated based on more specimens, when available.

*Lamellibrachia* sp. harbors dense populations of sulfur-oxidizing bacteria within its trophosome, as seen in other large siboglinid annelids. Sulfur granules seen in some bacteria indicate ability to store sulfur, which can buffer temporal variations in fluid emissions (Duperron et al. 2009). Trophosome sections looked circular, with a central lumen in the cylinder, surrounded by cells containing bacteria, which appeared larger the further away from the lumen. This resembles the structure of lobules documented from *Riftia pachyptila*, organized around an axial blood vessel, and a gradient from small rod-shaped to large coccoid-shaped bacteria toward the periphery (Bosch and Grasse 1984; Bright and Sorgo 2003; Katz et al. 2011). This organization was hypothesized to reflect progressive differentiation of host cells and growth of symbionts from center, with cells acting as stem cells, to the periphery of each lobule. Symbionts are very closely related to those of various *Lamellibrachia*, *Escarpia*, and *Seepiophila* species based on 16S rRNA, and amino acid sequence from APS reductase was identical to that of *Lamellibrachia* symbionts from the eastern Mediterranean (Duperron et al. 2009). RubisCO was also highly similar to a *Lamellibrachia* sp. symbiont sequence, but from Sagami Trough near Japan and not eastern Mediterranean (Elsaied and Naganuma 2001). Overall, the high level of similarity observed for all gene sequences with those from other Siboglinidae prevents any detailed investigation of symbiont biogeography, and new marker genes with higher levels of variations should be developed.

## Conclusion

Chemosymbiotic metazoans at the Guiness site harbor sulfur-oxidizing symbioses involving a dominant bacterial 16S rRNA phylotype in each species. Symbiont phylotypes are not shared among host species, or with other documented species, but these symbioses are very similar to symbioses documented in their respective groups. Symbionts contribute significantly to host nutrition, while organic matter from the downward flux of detritic material is probably not a major source. Gene analyses revealed heterogeneity in symbiont sequences in *Thyasira* sp. Guiness and *C. valdiviae*, and numerous incongruencies between 16S rRNA-based trees and trees based on other bacterial genes. This could illustrate occurrence of several copies of some genes, and recombinations of functional genes between strains of symbionts, or between symbionts and environmental bacteria. More robust databases of symbiont sequences for 23S RNA, APS reductase, and RubisCO are needed, and variability of symbiont lineages within a single host species should be investigated. None of the genes used here is alone appropriate for properly investigating symbiont biogeography, because of a lack of sequence variability. Multiple new markers need to be tested to better understand the biogeography and evolution of symbiosis in the metazoan groups investigated.

## Supporting Information

**Figure S1.** Organisms investigated in this study and sampling sites. *Acharax* sp. (A); *Thyasira* sp. with left valve removed (B), the gill is visible is pink; juvenile specimens of *Elenaconcha guiness* (C); adult specimen of *Calyptogena valdiviae* (D, photo by Ann Andersen); anterior part of *Lamellibrachia* sp. out of their tubes (E) and as seen in situ (G). The gill appears pinkish, the vestimentum is brown; Net 2 sampling site (F); net 3 sampling site (H).

**Figure S2.** Phylogenetic tree based on the analysis of a fragment of the RubisCO-encoding gene (117 amino acid positions analyzed). Sequences from this study are in bold. For methodology see Material anad Methods. Tree was rooted on several groups of Type I RubisCO, not shown. Bootstraps calculated from 500 NJ replicates (>60 shown) and Bayesian posterior probabilities (>0.85 shown) are displayed at nodes as “bootstrap/probability.” Scale bar represents 5% estimated sequence divergence.
