# Supplementary material for: Diversity of symbioses between chemosynthetic bacteria and metazoans at the Guiness cold seep site (Gulf of Guinea, West Africa)
Source: Microbiologyopen. 2012 Nov 21;1(4):467–80. doi: 10.1002/mbo3.47 (PMC3535391; doi:10.1002/mbo3.47)
Supplement: Supplementary file 1 [file mbo30001-0467-SD1.pptx]

## Slide 1
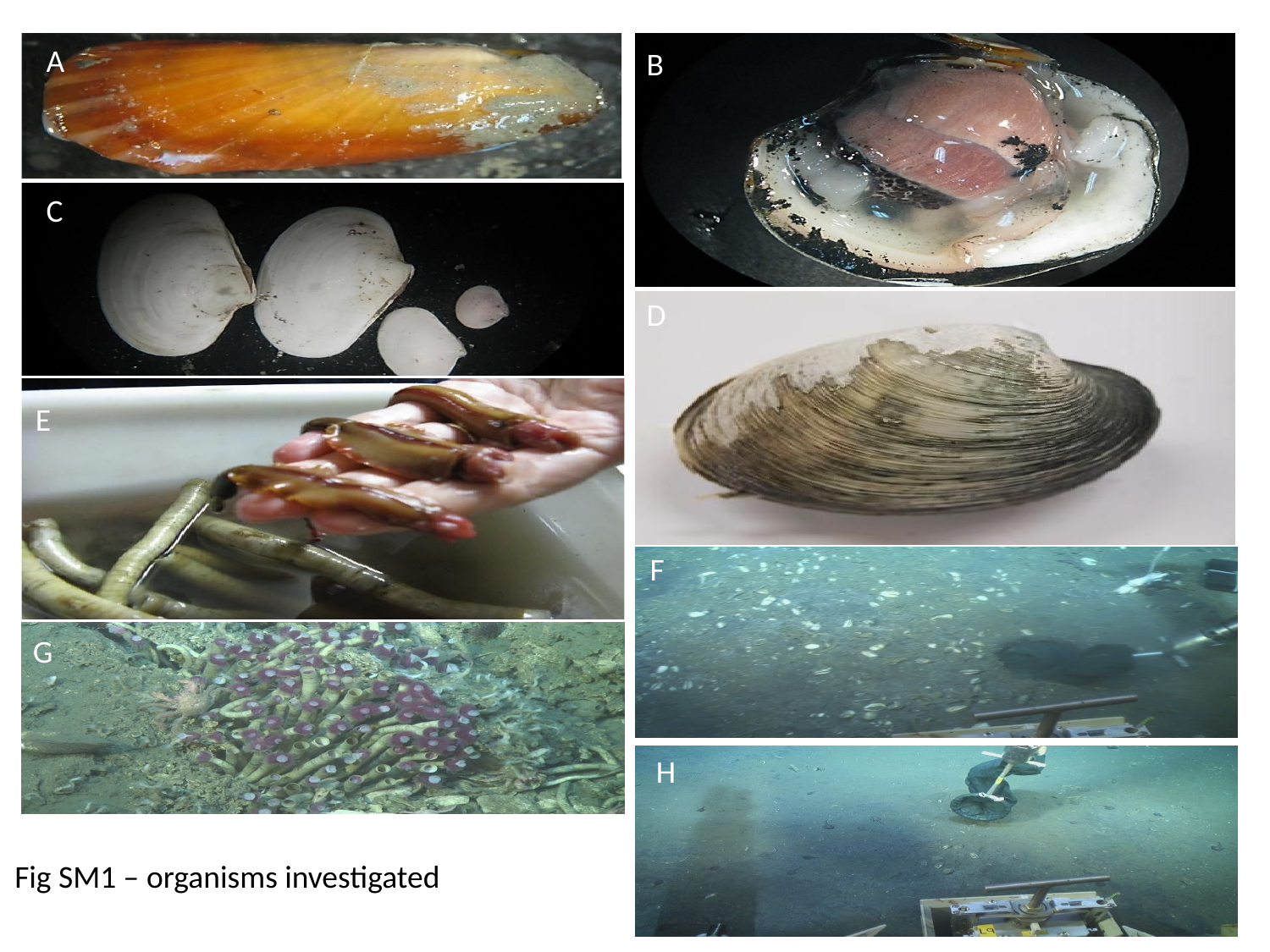

A
B
C
D
E
F
G
H
Fig SM1 – organisms investigated

## Slide 2
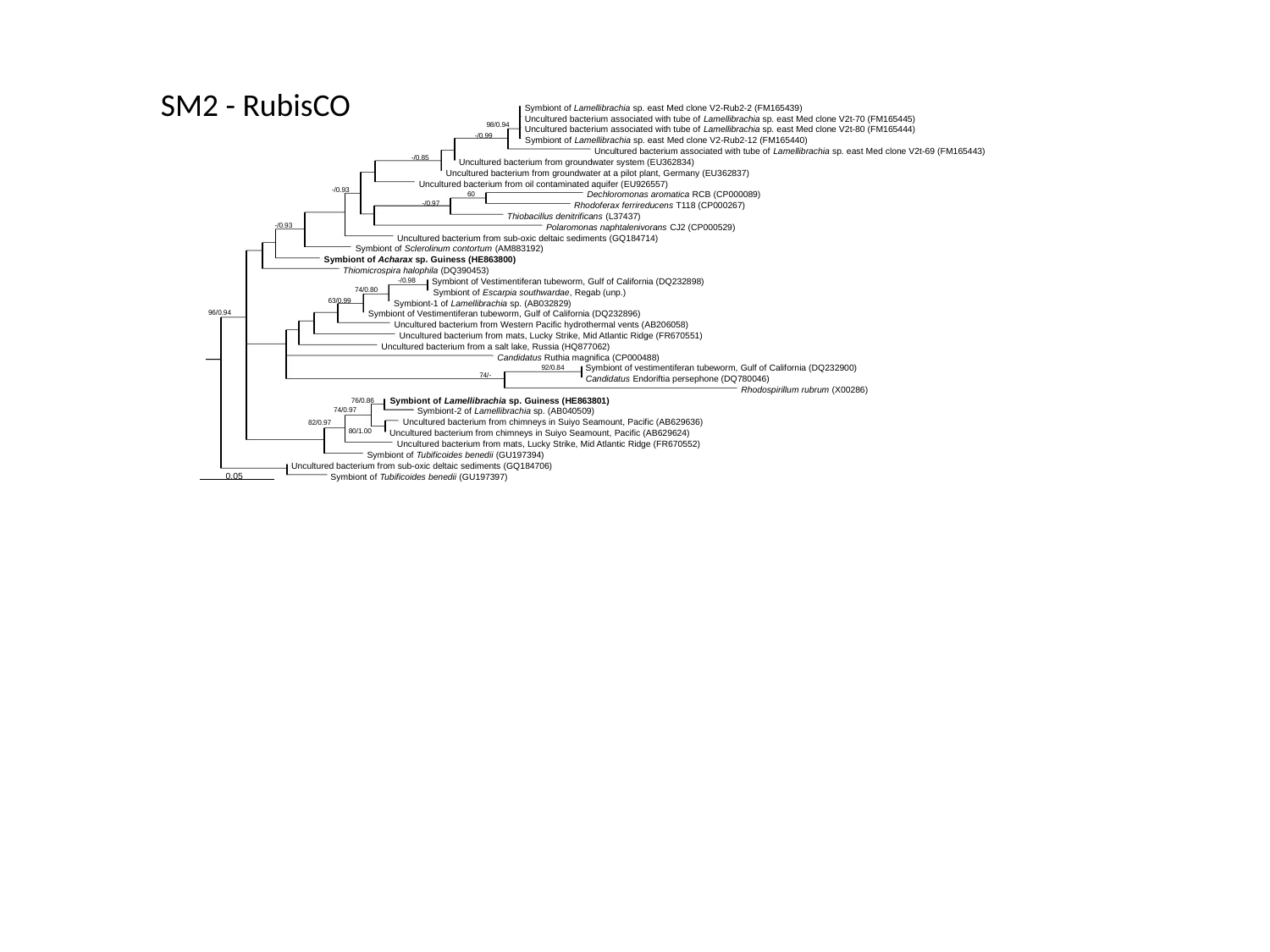

SM2 - RubisCO
Symbiont of Lamellibrachia sp. east Med clone V2-Rub2-2 (FM165439)
Uncultured bacterium associated with tube of Lamellibrachia sp. east Med clone V2t-70 (FM165445)
98/0.94
Uncultured bacterium associated with tube of Lamellibrachia sp. east Med clone V2t-80 (FM165444)
-/0.99
Symbiont of Lamellibrachia sp. east Med clone V2-Rub2-12 (FM165440)
Uncultured bacterium associated with tube of Lamellibrachia sp. east Med clone V2t-69 (FM165443)
-/0.85
Uncultured bacterium from groundwater system (EU362834)
Uncultured bacterium from groundwater at a pilot plant, Germany (EU362837)
Uncultured bacterium from oil contaminated aquifer (EU926557)
-/0.93
Dechloromonas aromatica RCB (CP000089)
60
-/0.97
Rhodoferax ferrireducens T118 (CP000267)
Thiobacillus denitrificans (L37437)
-/0.93
Polaromonas naphtalenivorans CJ2 (CP000529)
Uncultured bacterium from sub-oxic deltaic sediments (GQ184714)
Symbiont of Sclerolinum contortum (AM883192)
Symbiont of Acharax sp. Guiness (HE863800)
Thiomicrospira halophila (DQ390453)
Symbiont of Vestimentiferan tubeworm, Gulf of California (DQ232898)
-/0.98
74/0.80
Symbiont of Escarpia southwardae, Regab (unp.)
63/0.99
Symbiont-1 of Lamellibrachia sp. (AB032829)
96/0.94
Symbiont of Vestimentiferan tubeworm, Gulf of California (DQ232896)
Uncultured bacterium from Western Pacific hydrothermal vents (AB206058)
Uncultured bacterium from mats, Lucky Strike, Mid Atlantic Ridge (FR670551)
Uncultured bacterium from a salt lake, Russia (HQ877062)
Candidatus Ruthia magnifica (CP000488)
Symbiont of vestimentiferan tubeworm, Gulf of California (DQ232900)
92/0.84
74/-
Candidatus Endoriftia persephone (DQ780046)
Rhodospirillum rubrum (X00286)
Symbiont of Lamellibrachia sp. Guiness (HE863801)
76/0.86
74/0.97
Symbiont-2 of Lamellibrachia sp. (AB040509)
Uncultured bacterium from chimneys in Suiyo Seamount, Pacific (AB629636)
82/0.97
80/1.00
Uncultured bacterium from chimneys in Suiyo Seamount, Pacific (AB629624)
Uncultured bacterium from mats, Lucky Strike, Mid Atlantic Ridge (FR670552)
Symbiont of Tubificoides benedii (GU197394)
Uncultured bacterium from sub-oxic deltaic sediments (GQ184706)
0.05
Symbiont of Tubificoides benedii (GU197397)
